# A Girl’s Training

**Published:** 1890-09

**Authors:** 


### A GIRL'S TRAINING.

That portion of her life which a girl spends in her first home is incomplete and almost useless unless it includes a thorough and systematic training for the future. The probabilities are that she will be a wife and a mother one day, and her education must be judiciously directed to that end ; yet there are many of our girls who, if they do not remain single all their lives, are in that state when the old home is broken up, and they find themselves alone in a world in which they must struggle for a livelihood. A girl's training, then, must include also a preparation for that possibility, and the safest course is to give all our girls a fair chance of maintaining themselves if adversity overtakes them. It is well if parents are able to leave their children in comfort and independence, yet riches have been known to take to themselves wings and fly away.

Girls are not to blame in nine cases out of ten, if they are wild and reckless and good-for-nothing. If you buy a piano, you house it, and take excellent care of it. You never leave it out of doors, nor allow the children to cram its tuneful recesses with pins and pebbles. And yet it is only a piano, and represents merely a nominal valuation. It is not going to rise up and confront you one day in heaven, and demand to know, before God and high angels, why you did not take better care of it. It is not going to look at you with beseeching eyes from a dying bed, and ask you why you did not teach it to be pure and sinless and honest, that it might know whither it was going, out into the unknown dark. The girls will be troublesome witnesses to your neglect and indifferent care, when the piano is nothing but a heap of material wreck and ruin. You cannot slight your daughter and find your loss only a loss of the pocket-book. There is something in the mechanism of a girl's soul that will outlive a thousand Steinways, and demands far more delicate care and handling.

You cannot let your daughter run the streets, or make promiscuous acquaintances, or hang around depots, or flirt with strange men, without footing up a terrible bill of damages, that must be paid in tears rather than in currency. Having done your best to make a pleasant home, see that the girls are kept within it, or, if not, know where they are. The streets are full of irresponsible adventurers, whose aim is the ruin of happy homes. The idle tale of a worthless companion, the suggestion of an impure novel, may recruit an innocent girl into the worst of company.

There is no use in watching and protecting your girl spasmodically ; you cannot secure her from evil in that way, any more than you can keep your piano in tune by providing a tuner once in five years, and then allowing the keys to be pounded with a hammer in the interim. Protection and care must be constant and enduring, like the roof above your head and the foundation beneath your house.

The world is full of wolves thirsting for lamb's blood, and it behooves us to build a fold for the lambs, and see that we keep them in it.

